# The inhibition of mirror generalization of letters in school-aged children

**DOI:** 10.3389/fpsyg.2023.996012

**Published:** 2023-01-26

**Authors:** Deusa Priscila da Silva Resque, Adriany Maria de Moura Lobato, Carolina Gomes da Silva, Daniel Alves da Cruz Filho, Susanne Suely Santos da Fonseca, Felipe de Oliveira Matos, Antonio Pereira

**Affiliations:** ^1^Graduate Program in Neuroscience and Cell Biology, Institute of Biological Sciences, Federal University of Pará (UFPA), Belém, Brazil; ^2^Laboratory of Signal Processing, Department of Electrical and Biomedical Engineering, Institute of Technology, Federal University of Pará (UFPA), Belém, PA, Brazil; ^3^Department of Human Movement Sciences, State University of Maringá, Maringá, PR, Brazil

**Keywords:** literacy, mirror generalization, reading ability, mental rotation, human development, visuospatial, gender stereotyping, spatial cognition

## Abstract

Gender differences in spatial abilities favor males in both childhood and adulthood. During early development, this discrepancy can be attributed, among other things, to the influence of an early testosterone surge in boys, societal stereotypes, and expectations about gender. In the present work, we created a spatial task (including letter rotation and letter mirroring) which used letters as stimuli and evaluated the performance of school-aged children (6–10 years old). During this age period, children are being taught literacy skills which rely on the reorganization of cortical networks and the breakdown of mirror generalization. We divided our sample (*N* = 142, 73 females) into two age groups: 1^st^–2^nd^ (literacy acquisition; *N* = 70, 33 females) and 3^rd^–5^th^ (literacy consolidation; *N* = 72, 40 females) graders. While boys performed significantly better in letter rotation in the older group, girls’ performance remained substandard in both groups. This pattern is reversed for the mirror task, with older girls outperforming their younger counterparts and boys having similar performance in the two groups. Since the age period of our sample is not associated with large variations in the levels of reproductive steroids, we propose that the similarity of performance between younger and older girls in mental rotation of letters could be associated with society’s traditional attitudes and expectations on the relationship between visual–spatial skills and gender. As for the mirror task, while only girls had a significant difference between the two age groups, boys did show an improvement, as expected for the inhibition of mirror generalization for letters during reading acquisition.

## Introduction

Mirror generalization or mirror invariance describes the natural property of the visual system to recognize objects as being identical regardless of their spatial orientation (Rollenhagen and Olson, 2000). However, about 5,500 BCE, the invention of writing required the efficient discrimination of small orthographic signals which sometimes are mirror-symmetric, such as the letters b, d, p, and q in the modern Latin script. The selective inhibition of mirror generalization for orthographic processing is a pre-condition for reading proficiency and is acquired through effortful learning (Dehaene et al., 2005, 2010; Ahr et al., 2016). Reading acquisition depends on the rewiring and repurposing of a network of visual areas located in the inferior temporal cortex originally tasked with visual recognition of faces and objects. In particular, the *visual word form area* (WFA) is activated when orthographic strings are displayed in various writing systems in adults and children learning to read (Pegado et al., 2011; Dehaene-Lambertz et al., 2018). In fact, illiterates or readers of some scripts, such as Tamil or Thai, which do not have mirrored letters, have poor mirror discrimination (Nicole and Heather, 2015; Fernandes et al., 2021).

Mental rotation, defined as the ability to mentally retain and rotate abstract configurations in 2-D and 3-D space (Linn and Petersen, 1985), is an important spatial ability associated with many daily living activities (Newcombe and Frick, 2010). Mental rotation is also distinctly related to success in professional and academic careers in STEM (Science, Technology, Engineering, and Mathematics). Due to its role in STEM achievement (Bruce and Hawes, 2015), mental-rotation skills have been the target of much interest in education, including whether occasional gains obtained with training are transferable to other untrained skills (Cheng and Mix, 2014; Meneghetti et al., 2017; Cheung et al., 2020). During mental-rotation tasks, subjects are asked to judge stimuli that are presented in different orientations. Reaction times increase consistently with the angle between the target stimulus and a canonical orientation, suggesting that before judgment the stimulus is first mentally rotated into the canonical orientation (Cooper and Shepard, 1973).

Previous studies have shown that men and boys outscore women and girls in mental-rotation tasks involving abstract objects (Voyer et al., 1995; Geiser et al., 2008; Moè, 2009, 2018; Maeda and Yoon, 2013; Levine et al., 2016; Lauer et al., 2019; Lütke and Lange-Küttner, 2021). A popular explanation for this gap proposes that males and females may have experienced different selective pressures for specific spatial capacities during human evolution (Geary, 2022). However, more recent analyses propose that the male and female brain are not dimorphic and sex-related variances in brain’s structure and connectivity patterns are negligible and gender differences in cognitive abilities are probably associated with individual variance in genetic, epigenetic, and experiential factors (Eliot et al., 2021). A recent meta-analysis showed that a small male advantage in mental-rotation performance emerges during childhood and then subsequently increases with age, reaching a moderate effect size during adolescence (Lauer et al., 2019). While a recent study (Barel and Tzischinsky, 2018) using 3-D and 2-D stimuli showed that sex differences are not apparent in children, another study showed that 10-year-old boys outperformed girls in tasks with 3D cube figures rotated in depth (Ruthsatz et al., 2014).

Due to their ecological importance, letters have been used extensively in perception studies as visual stimuli. When deciding whether rotated letters are normal or mirror-reversed, subjects mentally rotate the letters into their canonical orientation, as with other visual stimuli, and then add a further step: flipping the letter along their vertical axis onto the canonical orientation (Cooper and Shepard, 1973; Corballis and McLaren, 1984; Corballis, 1988; Hamm et al., 2004). The importance of visual–spatial skills for reading acquisition is highlighted by the fact that dyslexic children are impaired in the mental rotation of letters, objects, and pictures (Rüsseler et al., 2005; Giovagnoli et al., 2016). However, to our knowledge, only one previous study, performed with second- and fourth-graders from Germany, used letters in mental-rotation studies aimed at comparing the performance of male and female schoolchildren (Neuburger et al., 2011). Besides their importance for understanding the role played by visual–spatial skills in reading acquisition, letters are also ecologically fit stimuli for studies aiming to evaluate the visuospatial abilities of school-aged children.

In the present study, we aim to contribute to the understanding of the developmental trajectory of gender differences in spatial aptitude by comparing the performance of school-aged children aged 6 to 10 years, grouped according to school year, in a computerized test requiring the mental rotation of letters. We devised a simple task that would be suitable to schoolchildren, easily applicable, and not time-consuming. The experimental paradigm was partially adapted from the letter-condition subtest proposed by Neuburger et al. (2011), with one target letter on the upper half of a screen side and four distractor stimuli on the bottom.

## Methods

### Participants

One hundred forty-two subjects ranging in age from 6 to 10 years were recruited and participated in the study at two public elementary schools located in Belém, Brazil (69 male, 7,9 ± 1,3 years and 73 female, 8,0 ± 1,4 years). The Brazilian Common Core Standards (*Base Nacional Curricular Comum*) establishes that basic literacy acquisition should be the focus of the first 2 years of elementary school (1^st^ and 2^nd^ grades), while in the last 3 years (3^rd^, 4^th^, and 5^th^ grades), students should consolidate reading ability. Thus, participants were allocated into four groups regarding gender (female and male) and literacy stage (acquisition and consolidation; Table 1). Parents or guardians provided written consent prior to testing. All study protocols were approved by the Ethics Committee of the State University of Maringá (UEM), Brazil (5.553.548). The exclusion criterion was the presence of a history of psychiatric illness and/or neurological disorders.

**Table 1:** Socio-demographic profile of subjects.

	Groups			
	Male		Female	
	1st–2nd grades (acquisition)	3rd–5th grades (consolidation)	1st–2nd grades (acquisition)	3^rd^–5^th^ grades (consolidation)
*N* (%)	37(26.1)	32(22.5)	33(23.2)	40(28.2)
Family Income (minimum wage)				
≤1	20(54.1)	1(3.1)	14(42.4)	8(20)
1–3	17(45.9)	31(69.9)	19(57.6)	32(80)
≥3	-	-	-	-
Degree of Instruction (family tutor)				
Illiterate	-	-	1(3)	-
Incomplete Elementary School	6(16.2)	-	3(9.1)	-
Complete Elementary School	4(10.8)	8(25)	9(27.3)	13(32.5)
Incomplete High School	3(8.1)	24(75)	5(15.2)	27(67.5)
Complete High School	21(56.8)	-	13(39.4)	-
Incomplete University Education	2(5.4)	-	1(3)	-
Complete University Education	1(2.7)	-	1(3)	-

In our sample, 70 (49.30%) of students were in the 1^st^ and 2^nd^ grades (acquisition) and 72 (50.70%) were in the 3^rd^, 4^th^, and 5^th^ grades (consolidation). Most of the students (99, 69.72%) belonged to families with a monthly income of 1–3 minimum wages and the maximum educational level of most of the parents was incomplete high school education (59, 41.56%; Table 1). The rotation and mirror tests had Cronbach’s alpha values of 0.73 and 0.74, respectively.

### Procedure

**Figure 1:**
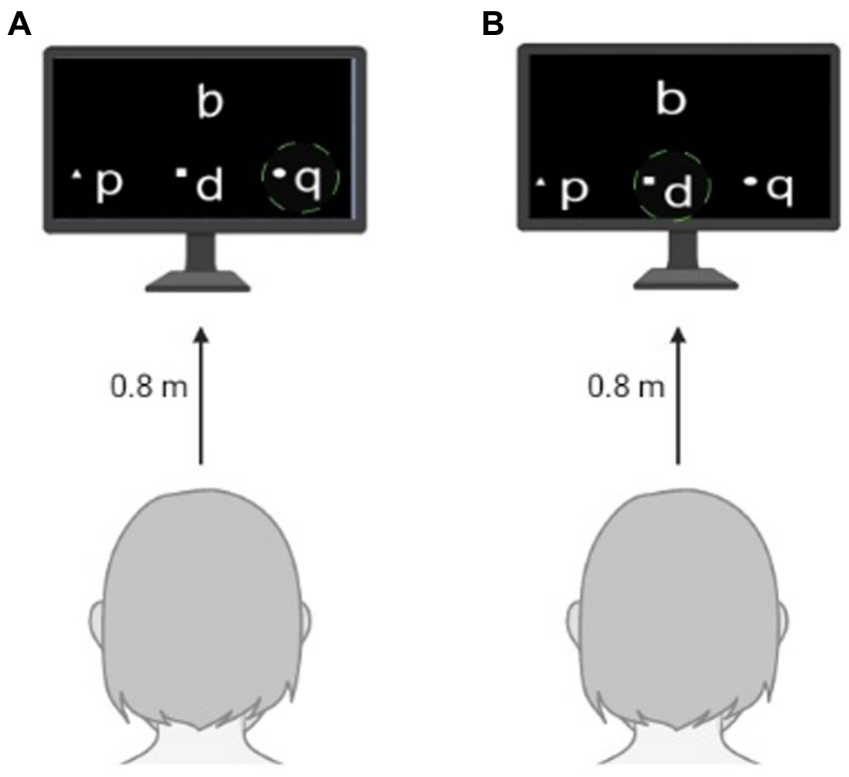
Experimental Setup for the letter rotation **(A)** and mirrored letter **(B)** tests. The green circle indicates the correct response (which was not shown to the participant).

Children were tested individually in a quiet location in their own schools with two computerized tasks adapted from the mental-rotation task proposed by Neuburger et al. (2011). In the rotation task, the letters q, p, d, and b were displayed on the computer screen in their canonical orientation and the participants should choose its rotated version from the remaining three letters presented simultaneously in a lower row on the same screen (Figure 1A). In the mirror task, a mirrored version of the letters q, p, d, or b was displayed on the computer screen and the participants should choose the correct answer from three alternatives presented simultaneously in a lower row on the screen (Figure 1B). The tasks were created and managed with the PsychoPy software (version 1.82.01, Open Science Tools Ltd.). Children sat opposite the experimenter in front of a computer screen (15.6 inches, resolution 1920 × 1080, refresh rate 60 Hz) located 0.8 m away from the participants. Participants looked at a fixed point on the screen and their hands were positioned on the keyboard. The following oral instruction was given before the rotation task: “*Welcome! You should indicate from the options below which letter corresponds to the letter located at the top of the display. Try to rotate the letters in your mind to find the better option. Please, try to respond as fast and accurately as you can. Thanks for your time!*.” For the mirror task, the instruction was: “*Welcome! You should indicate from the options below which letter is the mirror counterpart of the letter located at the top of the display. Please, try to respond as fast and accurately as you can. Thanks for your time!*” The participants performed 4 trials for each target stimulus, the response to each trial was coded either as correct (1) or incorrect (0) and subsequently added to obtain the total score.

### Statistical analysis

We performed a descriptive analysis with measures of central tendency and dispersion according to Gaussian distribution characteristics of the samples, verified through the Kolmogorov–Smirnov test. We used the Kruskal-Wallis test for performance comparisons among the four experimental groups (female-acquisition, female-consolidation, male-acquisition, and male-consolidation). The Mann–Whitney test with Bonferroni correction was used for the *post-hoc* comparison among groups. The internal consistency of the “rotation” and “mirror” tests was assessed using Cronbach’s Alpha. Statistical analyzes were performed with SPSS v.21 and the significance level was set at 95%. Statistical Power was verified *a posteriori* using Gpower (Faul et al., 2007).

## Results

**Figure 2:**
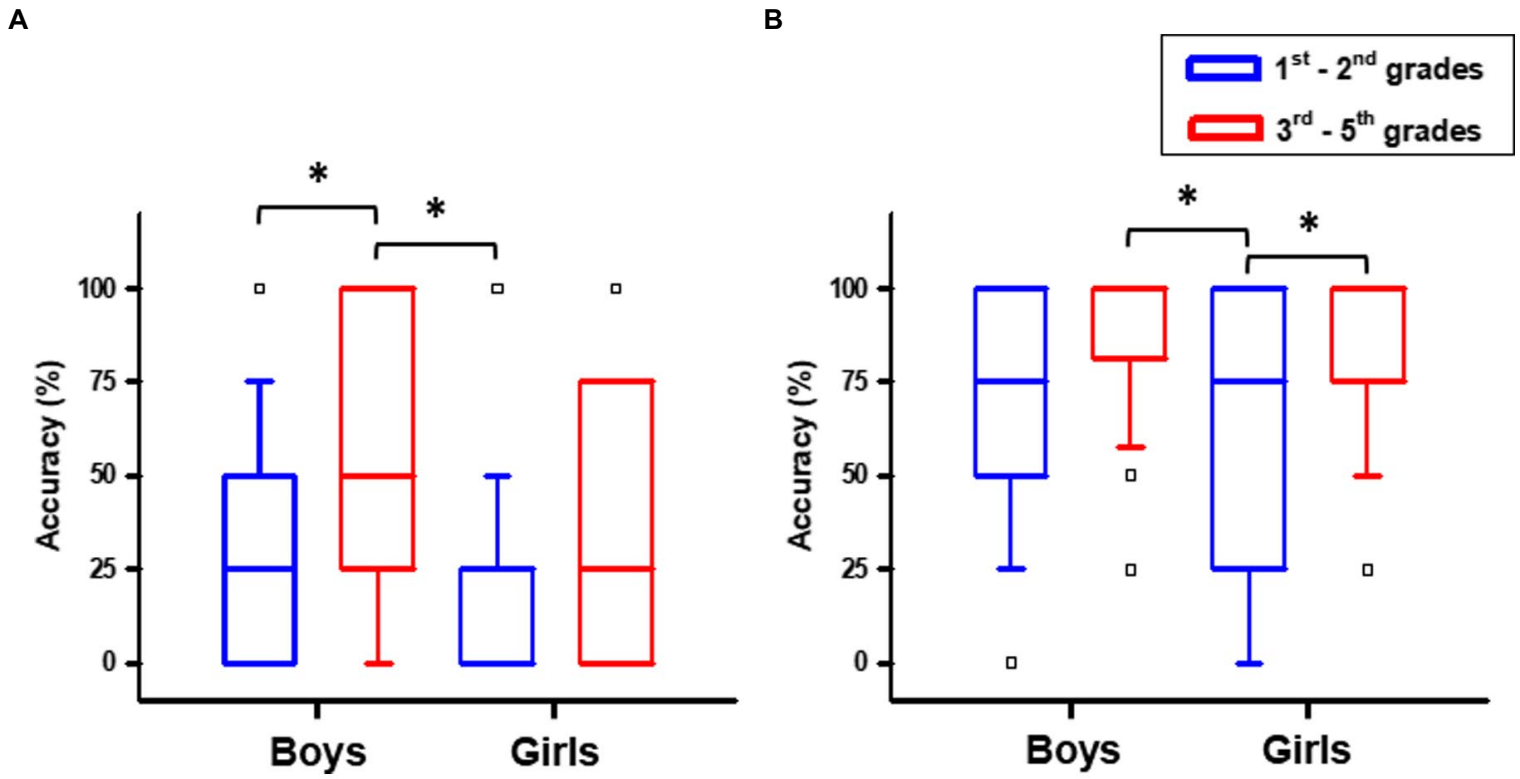
Test scores by age and sex: **(A)** rotation and **(B)** mirror task.

The Kruskal-Wallis test showed that groups differed on accuracy in the letter rotation test [H(3) = 15.595, *p* = 0.001] and the mirrored letter test [H(3) = 19.364, *p* < 0.001]. A Mann–Whitney *post-hoc* test with Bonferroni correction showed that the accuracy in the rotation task was higher for the male-consolidation group (Mdn = 50; IQR = 75) than for both the female- (Mdn = 0; IQR = 25; U = 37.124, Z = 3.786, *ps* = 0.001) and male- (Mdn = 25; IQR = 50; U = −25.362, Z = −2.658, *ps* = 0.047) acquisition groups. However, when the performance of boys and girls is compared in the consolidation stage, girls had lower performance (Mdn = 37.5; IQR = 75), though statistically indistinct from the other groups (*p* > 0.05; Figure 2A).

Regarding the mirror task (Figure 2B), girls in the acquisition group (Mdn = 75; IQR = 75) had lower scores than both boys (Mdn = 100; IQR = 19; U = 33.171, Z = 3.623 *ps* = 0.002) and girls (Mdn = 100; IQR = 25; U = −30.143, Z = −3.473, *ps* = 0.003) in the consolidation group. However, during acquisition, girls did not differ (*p* > 0.05) from boys (Mdn = 75; IQR = 50), with performance comparable to the consolidation groups.

**Figure 3:**
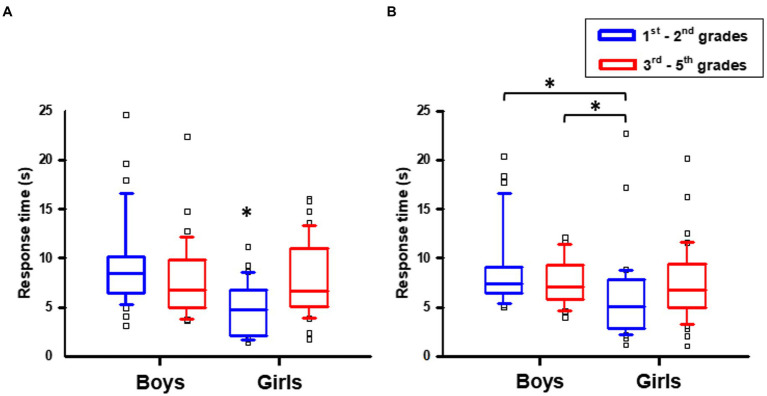
Response time by age and sex: **(A)** letter rotation test, **(B)** mirrored letter test.

The Kruskal-Wallis test showed that groups differed on response time both on the letter rotation [H(3) = 21.902, *p* < 0.001], and mirrored letter [H(3) = 18.143, *p* < 0.001] tasks (Figure 3). A Mann–Whitney *post-hoc* test with Bonferroni correction showed that the response time for the rotation task of the female-acquisition group (Mdn = 5.08; IQR = 5.08) was smaller than in the consolidation group, both male (Mdn = 6.75; IQR = 4.83; U = 27.415, Z = 2.686, *ps* = 0.043) and female (Mdn = 6.64; IQR = 5.90; U = −28.902, Z = −2.988, *ps* = 0.017), but not the male-acquisition group (Mdn = 8.40; IQR = 3.67; U = 45.7, Z = 4.64, *ps* < 0.001; Figure 3A).

As for the mirror task (Figure 3B), girls from the acquisition group (Mdn = 5.06; IQR = 4.91) had response times smaller than boys in the acquisition (Mdn = 7.33; IQR = 2.66; U = 39.783, Z = 4.039, *ps* < 0.001) and consolidation (Mdn = 7.07; IQR = 3.42; U = 32.899, Z = 3.224, *ps* = 0.008) groups. Performance of girls in the consolidation group (Mdn = 6.80; IQR = 4.39) was not statstically different from the other groups (*p* > 0.05), though.

## Discussion

Several previous findings show that visual–spatial abilities play a pivotal role in reading acquisition (Rüsseler et al., 2005; Giovagnoli et al., 2016). For instance, in early readers, the failure to inhibit the natural perceptual tendency to mirror generalization impairs the formation of adequate sound-letter correspondences and impedes fluent reading (for review, see Dehaene et al., 2015). This is evident, for instance, when early readers need to distinguish the words “dad” and “bad.”

One important visual–spatial ability is mental rotation, or the ability to transform a mental representation of an object to accurately predict how the object would look from a different angle. This is a skill we all use routinely when we try to mentally rearrange aspects of our visual world and which can be improved with training (Uttal et al., 2013; Hawes et al., 2015). However, the development of this ability is still poorly understood; including why, on average, adult males outperform adult females in mental-rotation tasks (Geiser et al., 2008; Moè, 2009; Maeda and Yoon, 2013; Levine et al., 2016).

A previous work tested German schoolchildren from the second and fourth grades with a paper–pencil mental-rotation task in three stimulus conditions (animal pictures, cube figures, and letters; Neuburger et al., 2011). The authors showed that fourth-grade boys and girls performed better than their second-grade colleagues in letter rotation (Neuburger et al., 2011). In the present study, we compared the performance of male and female subjects in a cross-sectional sample of male and female Brazilian Portuguese speakers in two distinct school groups associated with the acquisition and consolidation of reading ability. According to the official Brazilian literacy curriculum, children in the first, second, and third grades are taught basic literacy skills and children in the fourth and fifth grades are expected to consolidate earlier literacy gains toward reading proficiency. Our results show that both boys and girls performed poorly in the 1^st^–2^nd^ grade group, but only male students showed a significant improvement in the 3^rd^–5^th^ grade group. Response time of girls in the rotation task was particularly smaller in the 1^st^–2^nd^ grade group on both the mirror and rotation task, suggesting that they were guessing. Since the same students performed much better in the mirror task, we assume this reflects a selective limitation in their performance on mental-rotation tasks.

In a study using cubes, Titze et al. (2010) showed that a male advantage in mental-rotation scores was evident in a group with mean age of 10.3 ears but not in a younger group (mean age: 9.4 years). Still according to the Titze et al.’ (2010) study, performance of women, older girls, and younger girls did not differ significantly, whereas older boys and adults outperformed younger boys. This trend was recently confirmed by Rahe and Jansen (2022) in adolescents. Our results with the mirror task show that there is a trend for both boys and girls for the breakdown of mirror generalization for letters with the advance of their literacy instruction (Pegado et al., 2014). However, only girls reached the statistical criterion for difference between the acquisition and consolidation groups. Titze et al. (2010) proposed two hypotheses to explain gender differences in mental rotation in school-aged children: disparity in testosterone levels and self-expectations about performance. The influence of reproductive steroids on mental rotation has been studied for some time and has shown effects of both androgens (testosterone) and estradiol, with the former having a positive effect and the latter having a inhibitory one (Hampson, 2018). A recent study showed that the correlation between testosterone levels and mental-rotation performance is already evident in infants 5–6 months of age (Constantinescu et al., 2018). On the other hand, several studies have recently proposed that spatial anxiety and self-expectations in performance may also be important factors behind male superiority in mental-rotation tests (Moè, 2009; Alvarez-Vargas et al., 2020; Arrighi and Hausmann, 2022). Both characteristics can be exacerbated by social situational threats and gender-stereotyping still prevalent in modern societies (Lauer et al., 2019). For instance, the aforementioned study by Constantinescu et al. (2018) showed a negative correlation between parents’ gender-stereotypical attitudes and mental-rotation performance only in girls. Other research suggests that stereotype threats, or the worry of confirming a negative stereotype, may negatively influence intrinsic motivation and interest in stereotyped tasks and domains (Thoman et al., 2013; Doyle and Voyer, 2016). In other words, stereotype threats create a negative feedback loop that may push stigmatized groups from certain activities, careers, and academic pathways. Another important issue in this regard are the experiential differences in spatial-promoting activities, digital or not (Terlecki and Newcombe, 2005; Lauer et al., 2018), between boys and girls at this age. In summary, gender should not be construed as a causal parameter in comparisons between male and female cognitive performance, but as a surrogate for associated biological and environmental factors.

## Limitations

Both the rotation and the mirror task used only four test items: the letters q, p, d, and b, the ones which have mirror-image counterparts in the Latin alphabet. The tasks were designed to be applied together to allow a quick appraisal of the inhibition of mirror generalization processes in school children. Among the limitations, we could mention the lack of other types of stimuli items, such 3D cubes, 2D animals or objects, and letters without mirror-image counterparts. The accuracy of girls in the rotation task, both in the younger and the older group, was close to chance, suggesting that the task may be particularly difficult for girls in these age groups. However, these results could also reflect the selective role of environmental factors negatively affecting mostly girls.

## Data availability statement

The raw data supporting the conclusions of this article will be made available by the authors, without undue reservation.

## Ethics statement

The studies involving human participants were reviewed and approved by Ethics Committee of the State University of Maringá (UEM; 5.553.548). Written informed consent to participate in this study was provided by the participants’ legal guardian/next of kin.

## Author contributions

AP: analysis, conceptualization, funding acquisition, methodology, project administration, resources, supervision, and writing. FO: analysis, supervision, and writing. DR: investigation, analysis, and writing. AM, DC, and SF: investigation. All authors contributed to the article and approved the submitted version.

## Funding

This work was financially support by CNPQ (Research Productivity 312060/2020-3 for AP) and UFPA/PAPQ.

## Conflict of interest

The authors declare that the research was conducted in the absence of any commercial or financial relationships that could be construed as a potential conflict of interest.

## Publisher’s note

All claims expressed in this article are solely those of the authors and do not necessarily represent those of their affiliated organizations, or those of the publisher, the editors and the reviewers. Any product that may be evaluated in this article, or claim that may be made by its manufacturer, is not guaranteed or endorsed by the publisher.

## References

[ref1] Ahr E. Houdé O. Borst G. (2016). Inhibition of the mirror generalization process in reading in school-aged children. J. Exp. Child Psychol. 145, 157–165. doi: 10.1016/j.jecp.2015.12.009, PMID: 26827098

[ref2] Alvarez-Vargas D. Abad C. Pruden S. M. (2020). Spatial anxiety mediates the sex difference in adult mental rotation test performance. Cogn. Res. Princ. Implic. 5:31. doi: 10.1186/s41235-020-00231-8, PMID: 32712746 PMC7382671

[ref3] Arrighi L. Hausmann M. (2022). Spatial anxiety and self-confidence mediate sex/gender differences in mental rotation. Learn. Mem. 29, 312–320. doi: 10.1101/lm.053596.122, PMID: 36206394 PMC9488019

[ref4] Barel E. Tzischinsky O. (2018). Age and sex differences in verbal and visuospatial abilities. Adv. Cogn. Psychol. 14, 51–61. doi: 10.5709/acp-0238-x, PMID: 32362962 PMC7186802

[ref5] Bruce C. D. Hawes Z. (2015). The role of 2D and 3D mental rotation in mathematics for young children: what is it? Why does it matter? And what can we do about it? ZDM 47, 331–343. doi: 10.1007/s11858-014-0637-4

[ref6] Cheng Y.-L. Mix K. S. (2014). Spatial training improves Children’s mathematics ability. J. Cogn. Dev. 15, 2–11. doi: 10.1080/15248372.2012.725186

[ref7] Cheung C.-N. Sung J. Y. Lourenco S. F. (2020). Does training mental rotation transfer to gains in mathematical competence? Assessment of an at-home visuospatial intervention. Psychol. Res. 84, 2000–2017. doi: 10.1007/s00426-019-01202-5, PMID: 31144028

[ref8] Constantinescu M. Moore D. S. Johnson S. P. Hines M. (2018). Early contributions to infants’ mental rotation abilities. Dev. Sci. 21:e12613. doi: 10.1111/desc.12613, PMID: 29143410

[ref9] Cooper L. A. Shepard R. N. (1973). “Chronometric studies of the rotation of mental images,” in Visual Information Processing. ed. Chase W. G. (New York, NY: Academic Press), 135–142.

[ref10] Corballis M. C. (1988). Recognition of disoriented shapes. Psychol. Rev. 95, 115–123. doi: 10.1037/0033-295X.95.1.115 3281177

[ref11] Corballis M. C. McLaren R. (1984). Winding one’s Ps and Qs: mental rotation and mirror-image discrimination. J. Exp. Psychol. Hum. Percept. Perform. 10, 318–327. doi: 10.1037/0096-1523.10.2.318, PMID: 6232348

[ref12] Dehaene S. Cohen L. Sigman M. Vinckier F. (2005). The neural code for written words: a proposal. Trends Cogn. Sci. 9, 335–341. doi: 10.1016/j.tics.2005.05.004, PMID: 15951224

[ref13] Dehaene S. Pegado F. Braga L. W. Ventura P. Nunes Filho G. Jobert A.. (2010). How learning to read changes the cortical networks for vision and language. Science 330, 1359–1364. doi: 10.1126/science.1194140, PMID: 21071632

[ref01] Dehaene S. Cohen L. Morais J. Kolinsky R. (2015). Illiterate to literate: behavioural and cerebral changes induced by reading acquisition. Nature Rev. Neurosci. 16, 234–244. doi: 10.1038/nrn3924 25783611

[ref14] Dehaene-Lambertz G. Monzalvo K. Dehaene S. (2018). The emergence of the visual word form: longitudinal evolution of category-specific ventral visual areas during reading acquisition. PLoS Biol. 16:e2004103. doi: 10.1371/journal.pbio.2004103, PMID: 29509766 PMC5856411

[ref15] Doyle R. A. Voyer D. (2016). Stereotype manipulation effects on math and spatial test performance: a meta-analysis. Learn. Individ. Differ. 47, 103–116. doi: 10.1016/j.lindif.2015.12.018

[ref16] Eliot L. Ahmed A. Khan H. Patel J. (2021). Dump the “dimorphism”: comprehensive synthesis of human brain studies reveals few male-female differences beyond size. Neurosci. Biobehav. Rev. 125, 667–697. doi: 10.1016/j.neubiorev.2021.02.026, PMID: 33621637

[ref17] Faul F. Erdfelder E. Lang A.-G. Buchner A. (2007). G*Power 3: a flexible statistical power analysis program for the social, behavioral, and biomedical sciences. Behav. Res. Methods 39, 175–191. doi: 10.3758/BF03193146, PMID: 17695343

[ref18] Fernandes T. Arunkumar M. Huettig F. (2021). The role of the written script in shaping mirror-image discrimination: evidence from illiterate, Tamil literate, and Tamil-Latin-alphabet bi-literate adults. Cognition 206:104493. doi: 10.1016/j.cognition.2020.104493, PMID: 33142163

[ref19] Geary D. C. (2022). Spatial ability as a distinct domain of human cognition: an evolutionary perspective. Intelligence 90:101616. doi: 10.1016/j.intell.2021.101616

[ref20] Geiser C. Lehmann W. Eid M. (2008). A note on sex differences in mental rotation in different age groups. Intelligence 36, 556–563. doi: 10.1016/j.intell.2007.12.003

[ref21] Giovagnoli G. Vicari S. Tomassetti S. Menghini D. (2016). The role of visual-spatial abilities in dyslexia: age differences in Children’s Reading? Front. Psychol. 7:1997. doi: 10.3389/fpsyg.2016.01997, PMID: 28066311 PMC5174111

[ref22] Hamm J. Johnson B. W. Corballis M. C. (2004). One good turn deserves another: an event-related brain potential study of rotated mirror–normal letter discriminations. Neuropsychologia 42, 810–820. doi: 10.1016/j.neuropsychologia.2003.11.009, PMID: 15037059

[ref23] Hampson E. (2018). Regulation of cognitive function by androgens and estrogens. Curr. Opin. Behav. Sci. 23, 49–57. doi: 10.1016/j.cobeha.2018.03.002

[ref24] Hawes Z. Moss J. Caswell B. Poliszczuk D. (2015). Effects of mental rotation training on children’s spatial and mathematics performance: a randomized controlled study. Trends Neurosci. Educ. 4, 60–68. doi: 10.1016/j.tine.2015.05.001

[ref25] Lauer J. E. Ilksoy S. D. Lourenco S. F. (2018). Developmental stability in gender-typed preferences between infancy and preschool age. Dev. Psychol. 54, 613–620. doi: 10.1037/dev0000468, PMID: 29154646

[ref26] Lauer J. E. Yhang E. Lourenco S. F. (2019). The development of gender differences in spatial reasoning: a meta-analytic review. Psychol. Bull. 145, 537–565. doi: 10.1037/bul0000191, PMID: 30973235

[ref27] Levine S. C. Foley A. Lourenco S. Ehrlich S. Ratliff K. (2016). Sex differences in spatial cognition: advancing the conversation. WIREs Cogn. Sci. 7, 127–155. doi: 10.1002/wcs.1380, PMID: 26825049

[ref28] Linn M. C. Petersen A. C. (1985). Emergence and characterization of sex differences in spatial ability: a meta-analysis. Child Dev. 56, 1479–1498. doi: 10.2307/1130467, PMID: 4075870

[ref29] Lütke N. Lange-Küttner C. (2021). The magical number four in children’s mental rotation of cube aggregates. Dev. Psychol. 57, 211–226. doi: 10.1037/dev0001139, PMID: 33539129

[ref30] Maeda Y. Yoon S. Y. (2013). A meta-analysis on gender differences in mental rotation ability measured by the Purdue spatial visualization tests: visualization of rotations (PSVT:R). Educ. Psychol. Rev. 25, 69–94. doi: 10.1007/s10648-012-9215-x

[ref31] Meneghetti C. Cardillo R. Mammarella I. C. Caviola S. Borella E. (2017). The role of practice and strategy in mental rotation training: transfer and maintenance effects. Psychol. Res. 81, 415–431. doi: 10.1007/s00426-016-0749-2, PMID: 26861758

[ref32] Moè A. (2009). Are males always better than females in mental rotation? Exploring a gender belief explanation. Learn. Individ. Differ. 19, 21–27. doi: 10.1016/j.lindif.2008.02.002

[ref33] Moè A. (2018). Mental rotation and mathematics: gender-stereotyped beliefs and relationships in primary school children. Learn. Individ. Differ. 61, 172–180. doi: 10.1016/j.lindif.2017.12.002

[ref34] Neuburger S. Jansen P. Heil M. Quaiser-Pohl C. (2011). Gender differences in pre-adolescents’ mental-rotation performance: do they depend on grade and stimulus type? Personal. Individ. Differ. 50, 1238–1242. doi: 10.1016/j.paid.2011.02.017

[ref35] Newcombe N. S. Frick A. (2010). Early education for spatial intelligence: why, what, and how. Mind Brain Educ. 4, 102–111. doi: 10.1111/j.1751-228X.2010.01089.x

[ref36] Nicole R. Heather W. (2015). Mirror invariance - a comparison between Thai and Roman script readers. Front. Psychol. 6:28. doi: 10.3389/conf.fpsyg.2015.66.0002825713542

[ref37] Pegado F. Comerlato E. Ventura F. Jobert A. Nakamura K. Buiatti M.. (2014). Timing the impact of literacy on visual processing. Proc. Natl. Acad. Sci. U.S.A. 111, E5233–E5242. doi: 10.1073/pnas.1417347111, PMID: 25422460 PMC4267394

[ref38] Pegado F. Nakamura K. Cohen L. Dehaene S. (2011). Breaking the symmetry: mirror discrimination for single letters but not for pictures in the visual word form area. NeuroImage 55, 742–749. doi: 10.1016/j.neuroimage.2010.11.043 21111052

[ref39] Rahe M. Jansen P. (2022). Does mindfulness help to overcome stereotype threat in mental rotation in younger and older adolescents? Psychol. Res. doi: 10.1007/s00426-022-01666-y, PMID: 35302181 PMC9928811

[ref40] Rollenhagen J. E. Olson C. R. (2000). Mirror-image confusion in single neurons of the macaque inferotemporal cortex. Science 287, 1506–1508. doi: 10.1126/science.287.5457.1506, PMID: 10688803

[ref41] Rüsseler J. Scholz J. Jordan K. Quaiser-Pohl C. (2005). Mental rotation of letters, pictures, and three-dimensional objects in German dyslexic children. Child Neuropsychol. 11, 497–512. doi: 10.1080/09297040490920168, PMID: 16306024

[ref42] Ruthsatz V. Neuburger S. Jansen P. Quaiser-Pohl C. (2014). “Pellet figures, the feminine answer to cube figures? Influence of stimulus features and rotational axis on the mental-rotation performance of fourth-grade boys and girls” in Spatial cognition IX lecture notes in computer science. eds. Freksa C. Nebel B. Hegarty M. Barkowsky T. (Cham: Springer International Publishing), 370–382.

[ref43] Terlecki M. S. Newcombe N. S. (2005). How important is the digital divide? The relation of computer and videogame usage to gender differences in mental rotation ability. Sex Roles 53, 433–441. doi: 10.1007/s11199-005-6765-0

[ref44] Thoman D. B. Smith J. L. Brown E. R. Chase J. Lee J. Y. K. (2013). Beyond performance: a motivational experiences model of stereotype threat. Educ. Psychol. Rev. 25, 211–243. doi: 10.1007/s10648-013-9219-1, PMID: 23894223 PMC3719418

[ref45] Titze C. Jansen P. Heil M. (2010). Mental rotation performance and the effect of gender in fourth graders and adults. Eur. J. Dev. Psychol. 7, 432–444. doi: 10.1080/17405620802548214

[ref46] Uttal D. H. Meadow N. G. Tipton E. Hand L. L. Alden A. R. Warren C.. (2013). The malleability of spatial skills: a meta-analysis of training studies. Psychol. Bull. 139, 352–402. doi: 10.1037/a0028446, PMID: 22663761

[ref47] Voyer D. Voyer S. Bryden M. P. (1995). Magnitude of sex differences in spatial abilities: a meta-analysis and consideration of critical variables. Psychol. Bull. 117, 250–270. doi: 10.1037/0033-2909.117.2.250, PMID: 7724690

